# Follicular fluid content and oocyte quality: from single biochemical markers to metabolomics

**DOI:** 10.1186/1477-7827-7-40

**Published:** 2009-05-04

**Authors:** Alberto Revelli, Luisa Delle Piane, Simona Casano, Emanuela Molinari, Marco Massobrio, Paolo Rinaudo

**Affiliations:** 1 Reproductive Medicine and IVF Unit, Department of Obstetrical and Gynecological Sciences, University of Torino, Via Ventimiglia 3, 10126 Torino, Italy; 2 Department of Obstetrics, Gynecology, and Reproductive Sciences, University of California, San Francisco, USA

## Abstract

The assessment of oocyte quality in human in vitro fertilization (IVF) is getting increasing attention from embryologists. Oocyte selection and the identification of the best oocytes, in fact, would help to limit embryo overproduction and to improve the results of oocyte cryostorage programs. Follicular fluid (FF) is easily available during oocyte pick-up and theorically represents an optimal source on non-invasive biochemical predictors of oocyte quality. Unfortunately, however, the studies aiming to find a good molecular predictor of oocyte quality in FF were not able to identify substances that could be used as reliable markers of oocyte competence to fertilization, embryo development and pregnancy. In the last years, a well definite trend toward passing from the research of single molecular markers to more complex techniques that study all metabolites of FF has been observed. The metabolomic approach is a powerful tool to study biochemical predictors of oocyte quality in FF, but its application in this area is still at the beginning. This review provides an overview of the current knowledge about the biochemical predictors of oocyte quality in FF, describing both the results coming from studies on single biochemical markers and those deriving from the most recent studies of metabolomics

## Background

The assessment of oocyte quality is rapidly becoming one of the major objectives of embryologists in human in-vitro fertilization (IVF). In fact, although classical IVF does not include any process of oocyte selection and only embryos (sometimes zygotes) undergo selection, the general trend toward limiting embryo "overproduction" and the rapidly improving results of oocyte cryostorage, together with the restrictive legal rules introduced in some Countries, have challenged embryologists to identify criteria to select, among all oocytes retrieved, the best oocytes to inseminate.

Multiple methods of oocyte selection have been proposed. The study of oocyte morphology is very popular, being relatively quick and simple; however, it leads to identify more frequently "negative" than "positive" predictors of oocyte quality, and overall is not fully satisfactory (for a comprehensive review: [1]). The same concepts may be applied to studies on polarizing microscopy analysis (for a review: []. Some authors have studied the expression of genes in the granulosa cells or in the oocyte itself, looking for specific molecular markers of oocyte quality (see the review [2]). Polar body biopsy is somewhere used to screen oocytes with chromosomal defects deriving from errors in the two meiotic divisions (see the review [3]). Most of these techniques are quite complicated, require expensive laboratory equipment and time-consuming procedures, and consequently are not currently applicable in the clinical practice.

Follicular fluid (FF) provides a very important microenvironment for the development of oocytes. FF is a product of both the transfer of blood plasma constituents that cross the blood follicular barrier and of the secretory activity of granulosa and thecal cells [4]. It is reasonable to think that some biochemical characteristics of the FF surrounding the oocyte may play a critical role in determining oocyte quality and the subsequent potential to achieve fertilization and embryo development. The analysis of FF components may also provide information on metabolic changes in blood serum, as the circulating biochemical milieu may be reflected in the composition of FF [5].

FF is easily available as it is aspirated together with the oocyte at the time of OPU. In the last years, the study of FF content has been conducted by assaying one or more substances in the fluid derived from individual follicles and by relating it (them) to the fate of the egg coming from that specific follicle. For study purposes, in order to correlate FF substances with oocyte quality, it is imperative that each individual follicle is aspirated individually. The procedure of separated, single follicle aspiration is uncomfortable both for the patient and for the physician because multiple vaginal punctures are required, with increased risk of vaginal bleeding and increased patient discomfort. Moreover, needle flushing with culture medium after every follicular puncture has to be performed in order to be sure that the oocyte is effectively retrieved from each specific follicle; flushing must be made with standard volumes of buffered solution in order to obtain a known dilution of FF substances. Another relevant problem in studies estimating oocyte quality in relation to FF composition is that oocyte nuclear maturity is assessed shortly after oocyte recovery only during ICSI cases. Oocyte maturity in IVF is usually assessed only the following day, and a mature oocyte could have completed the nuclear maturation when cultured. This could bias the results. Finally, it is not completely clear if the FF concentration of a given substance is a variable related to the quality of the follicle (and therefore possibly reflecting the quality of the oocyte inside it) or to the clinical characteristics of the patient, such as age, smoking habit or type of ovarian stimulation.

Overall, a clear correspondence between specific FF biochemical characteristics and measurable oocyte quality-linked, embryo-related variables has not been established to date. In the last years, the research in this area has progressed toward a more complex type of molecular analysis, metabolomics, that is the analysis of all substances contained in a biological fluid. This review provides an overview of the current knowledge about the biochemical predictors of oocyte quality in FF, describing both the results coming from studies on single biochemical markers and those deriving from the most recent studies of metabolomics. The chemical constituents of FF have been grouped in the following categories: a) hormones; b) growth factors of the Transforming Growth Factor-beta (TGF-beta) superfamily; c) other growth factors and interleukins; d) Reactive Oxygen Species (ROS); e) anti-apoptotic factors; f) proteins, peptides and amino-acids; g) sugars; h) prostanoids.

### Hormones

#### Gonadotropins

Intrafollicular concentrations of FSH and LH are affected by their circulating levels: in IVF cycles the serum levels are determined by the amount of exogenously administered gonadotropins and by the degree of pituitary suppression (relevantly reducing the endogenous gonadotropin secretion).

High FF concentrations of FSH [6], hCG [7], and LH [8] have been reported to promote oocyte maturation and to be associated with eggs having a high chance of fertilization. These findings are confirmed by immunohistochemistry studies that stained granulosa cells for hCG: oocytes that subsequently fertilized had, in fact, significantly more granulosa cells immunobound to hCG than non-fertilizable oocytes [9]. FF LH was observed to be consistently higher in follicles containing oocytes that resulted in embryos leading to successful IVF attempts [10].

It appears that gonadotropins play an important role in the secretion of several substances by granulosa cells (e.g. hyaluronic acid), in turn affecting oocyte development and maturation. They may also act synergistically with estradiol (E2) in enhancing cytoplasmic maturation of the egg and, via cyclic AMP (cAMP) secretion, control oocyte meiosis: higher levels of gonadotropins would improve these processes and lead to better oocytes, better embryos and improved pregnancy rate. Notably, GH seems to play a similar (and may be synergic with gonadotropins) role [10].

#### Growth hormone (GH)

GH is known to enhance the FSH-dependent E2 production by granulosa cells [11] as well as the formation of FSH and LH receptors in these cells [12]. GH synthesis occurs also in the follicle [13], thus GH could act sinergically with gonadotropins increasing the "estrogenicity" of the follicle microenvironment, in turn leading to better oocytes.

Although GH has been identified in FF, a clear association between intrafollicular GH levels and pregnancy has not been found. Mendoza [10] found a higher GH concentration in follicles containing oocytes that generated embryos leading to successful IVF attempts, but Tarlatzis [14] found the opposite: lower GH concentration in FFs from IVF pregnant women when compared to non-pregnant patients.

#### Prolactin (PRL)

PRL content was reported to be higher and cAMP lower in the FF of fertilized oocytes as compared to unfertilized eggs [15]. A link between high PRL [16] and low cAMP [17,18] levels in FF, fertilization and successful pregnancy, was observed in some studies, but was not confirmed by others [19-23]. Therefore, at present FF PRL is not considered a reliable marker of oocyte quality.

#### Estrogens, progesterone (P) and androgens

It is well known that a predominantly intrafollicular estrogenic environment is associated with good follicular growth and has anti-atresia effects. In addition, E2 enhances the cytoplasmic maturation of oocytes via a direct non-genomic action at the plasma membrane level, in turn inducing extracellular calcium influx into the cell and a specific pattern of Ca2+ oscillations [24].

Elevated E2 and E2/P ratio in FF indicate a more advanced stage of oocyte maturation and have been repeatedly found to be associated with a higher chance of achieving pregnancy [14,17,19,22,25-29]. This observation, however, was not confirmed by other studies [6,20,23,30,31].

There is also conflicting evidence regarding the meaning of progesterone levels in FF. Several authors found that a high FF P concentration (or a low E2/P ratio) was predictive of subsequent implantation and pregnancy [32-35], and considered it as a reflection of progressive follicle luteinization and reduction of aromatase activity linked to the achievement of the final egg maturation. On the other side, however, oocytes from follicles having high FF P were frequently found in association with postmature oocytes that fertilized abnormally and gave rise to multipronuclear embryos [36]. It seems that while an optimal exposure to P has positive effects over oocyte characteristics, an excessive exposure leads to a rapid worsening of the cell's quality; a clear knowledge of the threshold at which P begins to damage the oocyte is presently lacking.

Elevated FF androgen levels (testosterone) were associated with lower quality oocytes, and in particular with oocytes showing a trend toward lower cleavage rates after fertilization [37]. E2/T ratio was also reported to be higher in pregnancy-associated follicles [38,39]. Taken together, these data seem to indicate that a low estrogen/androgen ratio in FF may be associated with early follicular atresia, which negatively affects the viability of the enclosed oocyte and limits the chances of fertilization and pregnancy. Indeed, the notion that a predominantly androgenic intrafollicular environment may lead to follicular atresia is well accepted, but at the same time it is widely accepted that a certain amount of intrafollicular androgens is needed to obtain optimal follicular growth. In fact, in IVF cycles with unsatisfactory ovarian response to FSH, addition of LH (and thus stimulation of androgen synthesis by theca cells) has been found to improve follicle growth and oocyte maturation [40,41].

#### Corticoids

Corticoids in the FF have been supposed to be important to achieve final oocyte maturation and subsequent embryo implantation by authors that observed that a high FF cortisol/cortisone ratio was associated with the likelihood to become pregnant in IVF [42,43].

Interestingly, adjuvant corticoid therapy during IVF was advocated to promote a better ovarian response and the retrieval of more mature oocytes [44]. However, a recent paper in which prednisolone was administered in association with acetylsalicylic acid as an adjuvant treatment during IVF cycles failed to detect any advantage in terms of oocyte fertilization, embryo implantation and pregnancy in treated women [45].

### Growth factors of the Transforming growth factor-beta (TGF-beta) superfamily

#### Inhibin and Activin

Inhibins are produced by granulosa cells and their FF level reflect the number and activity of the granulosa cells of each follicle; in the FF, inhibin A increases while inhibin B decreases during the follicular phase [46,47]. FF inhibin A levels are significantly higher in patients with endometriosis [48,49].

A recent prospective study showed that higher levels of inhibin A and B were associated with the likelihood to retrieve oocytes at the time of OPU, but not with oocyte quality and fertilization competence [47]. Similarly, Fried [50] observed that inhibin B in FF and serum was strongly correlated to the number of oocytes retrieved, but not with the IVF outcome, so that inhibin could be considered as a marker of ovarian response, but not of oocyte or embryo quality.

Chang [51] found that inhibin B in FF may serve as an effective marker of follicular development; he also showed a significant correlation between inhibin B levels in FF and embryo scores on days 2 and 3, so he considered inhibin B a useful predictor of quality of the embryo. Another study reached similar conclusions: Ocal [52] found that high levels of inhibin A and B in the FF were associated withy increased fertilization and pregnancy rates.

Lau [46], on the other hand, found a positive correlation between good quality oocytes and FF levels of Activin A, but not with inhibins A and B. His researches were not further developed, so the role of Activin A as a marker of oocyte quality and its relationship with inhibin family needs further investigation.

#### Anti-mullerian hormone (AMH)

Data on AMH levels in relation to oocyte quality are still contradictory. Serum levels of AMH between 1.66 and 4.52 ng/mL-1 were found to be associated with high quality oocytes [53] and with good morphology embryos [54]. Cupisti [55] found that AMH levels in individual follicles were inversely correlated with the maturation and developmental potential of oocytes. On the contrary, Takahashi [56] observed that oocytes were more likely to be fertilized when their follicle was able to produce high levels of AMH, as FF AMH levels from follicles with fertilized oocytes were more than 3 times higher than from follicles with non-fertilized eggs.

#### Bone morphogenetic protein-15 (BMP-15)

BMP-15 was found to influence oocyte maturation and quality. Higher BMP-15 levels were observed in the FF of eggs that fertilized and cleaved in comparison with the unfertilized or uncleaved [57]. In the same study, intrafollicular BMP-15 levels were also shown to be positively correlated to FF estradiol levels in individual follicles. These results still need to be confirmed in larger studies.

### Other growth factors and interleukins

#### Insulin-like growth factors (IGF)

Insulin-like growth factors I and II (IGF-I and -II) are polypeptides that promote cell proliferation and differentiation in several tissues; their biological availability is regulated by a family of IGF-binding proteins (from IGFBP-1 to IGFBP-6).

The intrafollicular levels of IGF-II, IGFBP-3 and IGFBP-4 were found to be significantly correlated with oocyte fertilization, cleavage, embryo development and embryo morphological score on day 3 [58]. In the same study, multiple regression analysis showed that the combination of high FF levels of IGFBP-3 and 4 with low FF levels of pregnancy-associated plasma protein-A (PAPP-A) was significantly correlated with fertilization and embryo development.

In other studies, FF levels of both IGF-I and IGFBP-1 were positively correlated with oocyte quality and maturity [28,59,60], and the ratio IGF-1/IGFBP-1 was shown to be significantly higher both in serum and in FF in women whose IVF was successful [50].

In a recent study, however, IGF-I FF levels were not found to reflect embryo quality and IVF outcome [61]. Overall, further studies are needed to estabilish if IGFs and IGF-BPs have the potential to become useful non-invasive biomarkers of oocyte quality in the clinical practice.

#### Other growth factors

The growth factor amphiregulin, that probably mediates some of the effects of hCG on oocyte maturation, was recently detected in FF and its levels were inversely related to the fertilization rate; however, they were not associated with embryo quality [62].

Similarly in another study, no correlation with embryo quality and IVF outcome was observed for tumour necrosis factor-alpha (TNF-alpha), epidermal growth factor (EGF), and basic fibroblast growth factor (BFGF) [61].

#### Interleukins

Pro-inflammatory cytokines can be found in FF as a result of ovarian local synthesis and release during follicular maturation and ovulation; for example, FF IL-1beta derives both from the plasma ultrafiltrate and from the local synthesis by luteinizing granulosa cells.

A positive correlation was observed between the serum concentrations of IL-1beta and E2 on the day of hCG injection [63]. Higher FF IL-1beta levels were associated with normal fertilization [64], but surprisingly they were lower in FFs whose oocytes were able to generate better embryos and successful IVF attempts [10]. It is possible that IL-1beta leads to cytoplasmic maturation and normal fertilization, but does not play a role in post-fertilization embryo development.

In some studies, IL-2 and IL-10 were found to correlate with specific hormonal milieus within the follicle, but not with IVF outcome [65-67]. Ledee [68] recently detected significantly higher levels of IL-2 and interferon (IFN-gamma) in FFs whose oocytes generated early cleaving embryos. In the same study, IL-12 levels were higher in the FF of follicles corresponding to highly fragmented embryos, and granulocyte colony-stimulating factor (G-CSF) was particularly elevated in the fluid of follicles corresponding to embryos with high implantation potential.

Bili [69] studied the FF levels of IL-1alpha, IL-2, TNF-alpha and leukotriene B4 (LTB4) and reported that there was no significant relationship between their individual FF concentration and oocyte maturity, fertilization and likelihood of pregnancy. On the other hand, IL-1alpha/TNF-alpha, IL-1alpha/LTB4, TNF-alpha/LTB4 ratios were significantly different in the FF of the women who became pregnant versus the ones who did not. He suggested that IL-1alpha, TNF-alpha and LTB4 may take part in the process of follicle wall degradation, and their concentration in optimal proportion may reflect a better intrafollicular milieu able to optimize oocyte development and maturation.

### Reactive Oxygen Species (ROS)

#### ROS and antioxidant factors

Follicular vascularity, intrafollicular oxygen content and mitochondrial activity are factors sustaining an optimal oocyte development [70-72]. Studies on follicular vascularization and oxygenation may provide information on the developmental competence of the respective oocytes.

Oxidative stress may induce DNA damage and trigger apoptosis. In fact, severely hypoxic follicles contain oocytes with a high frequency of abnormalities of the meiotic spindle, with subsequent increased probability of aneuploidy in the embryo [73]. Hypoxic follicles that often contain oocytes with cytoplasmic defects give often rise to embryos with multinucleated blastomeres [73].

The impact of oxidative stress on oocyte maturation seems to be deleterious, although its exact role is not fully clarified and, in fact, conflicting results exist.

Surprisingly, some studies found a positive correlation between FF ROS levels and maturation parameters. ROS were reported to improve bovine oocyte developmental potential during in vitro maturation [74], and Attaran [75] reported that women who become pregnant in IVF had significantly higher ROS levels in the FF than non-pregnant patients. On the other side, a supraphysiological amount of ROS leading to oxidative stress is involved in the aetiology of defective embryo development [76] and it was reported that low levels of ROS in FF might represent a potential marker for predicting success in IVF [75].

It is likely that an optimal balance between oxygen available to the oocyte and antioxidants is critical to permit normal meiotic spindle formation and correct chromosome alignment; in fact, in the mouse model correct chromosomal alignment was found in follicles with high oxygen and antioxidant levels [77].

Overall, high ROS levels reflect a wide range of pathological conditions that affect fertility potential. The deleterious effect of ROS can be counteracted by scavengers of free radicals or by endogenous antioxidants, such as the enzymes superoxide dismutase (SOD) and selenium-dependent glutathione peroxidase (SeGPx). The presence of SOD was reported in human FF, and high FF SOD concentrations were associated with oocytes that failed to fertilize [78]. In contrast, high levels of SeGPx were found in follicles that yelded fertilizable oocytes, and lower levels were related to fertilization failure [79].

Also the pineal hormone melatonin was found to exert an antioxidant effect protecting oocytes from oxidative stress; its intrafollicular concentration was shown to be inversely correlated with that of the oxidation marker 8OH-deoxyguanosine; moreover, the administration of 3 mg/day oral melatonin improved fertilization rate [80].

Overall, the FF total antioxidant capacity (TAC) was found to be significantly higher in follicles bearing oocytes competent for fertilization than in follicles having non-fertilizable oocytes [81]. TAC, a measurable marker of antioxidant activity, showed a positive correlation with embryo quality and with pregnancy rates in IVF [82]. The presence of higher TAC amounts in FF than in serum probably reflects the antioxidant activity of granulosa cells [79]. Elevated TAC levels would be a marker of mature follicles leading to the growth of high-quality oocytes.

#### Nitric oxide (NO)

Granulosa cells contain the endothelial isoform of NO synthase (eNOS) and actively synthesize NO [83]. NO is a very unstable gas molecule with a very short half-life and it is hardly measurable with direct methods. However, it is rapidly transformed into nitrites and nitrates, which, in turn, may be measured in biological fluids.

FF levels of nitrites and nitrates were observed to be significantly lower in follicles containing mature oocytes that fertilized and generated dividing embryos (> 6 cells stage) [84]; conversely, higher follicular levels of the same molecules were found to be associated with extended fragmentation and lower implantation potential of embryos [85]. Furthermore, NO concentrations in FF were observed to be significantly higher in patients with endometriosis or hydrosalpinx [83], conditions that are both associated with oocytes of lower fertilization potential. Interestingly, an up-regulation of serum NO was associated with implantation failure in patient with tubal or peritoneal factor infertility [85].

Taken together, these data suggest that an excessive production of NO in the microenvironment surrounding the oocyte could trigger apoptosis within the follicle before fertilization, thus affecting oocyte development. FF concentrations of nitrites/nitrates could eventually be used a negative markers of oocyte quality; however, their assessment is rather tricky and their intrafollicular levels were not found to correlate with IVF outcome [86]; thus, it is unlikely that the measurement of NO or its derivates will be used to identify optimal oocytes.

#### Vascular Endothelial Growth Factor (VEGF)

VEGF has a potentially important role affecting perifollicular angiogenesis and regulating intrafollicular oxygen levels. FF VEGF levels are significantly correlated with the grade of perifollicular vascularity [87].

A prospective study reported that FF VEGF concentration was significantly higher when the oocyte failed to fertilize [88]; furthermore, high FF VEGF levels have also been associated with poor embryo morphology [89] and poor conception rates in IVF [73].

The reason why VEGF FF levels appear to be negative markers of oocyte quality could be the activation of VEGF synthesis in response to hypoxia within the oocyte-cumulus complex. An hypoxic status at this level could be responsible on one side for the high intrafollicular concentrations of the peptide, on the other side for the low quality of the oocytes. In IVF programs that include oocyte selection, VEGF level in FF could be used as an index to exclude oocytes that developed in an hypoxic follicle, but until now FF VEGF assay, that is time-consuming and expensive, has not been introduced in the clinical practice.

### Anti-apoptotic factors

Apoptotic changes in the follicle's microenvironment can affect IVF outcome: high levels of apoptotic granulosa cells were associated with low quality oocytes, whereas low levels were associated to embryos of good quality [90]. The incidence of apoptosis in cumulus granulosa cells was found to be significantly higher in older women, and lower in follicles of younger IVF patients who become pregnant; moreover, comparing granulosa cells of a single follicle to the IVF outcome of the corresponding oocyte, embryo quality showed a negative correlation with the level of cumulus cells apoptosis [91].

The activation of specific cellular pathways, such as those involving Tumor Necrosis Factor (TNF) and Fas-ligand (Fas-L), is very important in determining apoptosis within the follicle; even the oocyte itself expresses some receptors that are specific of the apoptotic pathways, such as TNF-R [92] and Fas [93].

Both soluble FAS (sFas) and its ligand sFas-L have been detected in FF where they probably regulate both follicular atresia and oocyte maturation during folliculogenesis; it has been reported that sFas concentrations are significantly higher in FFs containing mature oocytes [94,95], whereas low sFas and high sFas-L concentrations are related to high apoptosis level in the follicle and poor oocyte quality [96].

Nevertheless, when sFas was measured in the oocyte-cumulus colture medium its concentration was inversely related to embryo quality [95], and both sFas and sFas-L concentrations in FF of patients with PCO were not significantly different between patients who achieved or not pregnancy, demonstrating a lack of predictive value towards oocyte quality [97]. The overall analysis of data indicates that sFas concentration in FF is not a reliable marker of the quality of the oocyte and of the embryo obtained from it.

### Proteins, peptides and amino-acids

FF contains several proteins that derive from blood plasma or are secreted both by granulosa and thecal cells [98]. The analysis of protein composition of FF aimed at identifying molecules that could be used as markers of good follicular development in order to optimize oocyte selection processes [99].

The study of proteinic components in FF have been made by detecting specific proteins and correlating them to oocyte quality. In the last years, more complex molecular techniques have been introduced; they allow to simultaneously study dozens of proteins and peptides in a biological fluid and allow the so-called "proteomics".

The major problem in the proteomic analysys of complex biological fluids is that their protein composition is very complicated and dynamic; as a consequence, sensitive and high-resolving protein separation techniques are needed. The most popular approach to FF proteomics is presently based on two-dimensional gel electrophoresis followed by protein digestion and mass spectrometry [100]. Recently, easier techniques based on protein prefractionation by isoelectric focusing and subsequent nanoliquid chromatography and mass spectrometry have been proposed and applied to the study of FF proteins [101]. Overall, however, the general understanding about proteins and their role in follicular growth and oocyte maturation is still quite limited.

#### Alpha-Fetoprotein, CEA and CA-125

FF levels of alpha-Fetoprotein (alpha-FP), CEA and CA-125 were not found to change significantly from fluids containing oocytes able to be fertilized or not [102]. Different follicles of the same patient showed a wide range in the concentrations of the three antigens and none of them was related to oocyte quality [103]. In another study, CA-125 intrafollicular levels were not found to be related either to a specific follicular hormone or to the outcome of the IVF cycle [104].

#### Antigen CD44

CD44 is a membrane-integrated protein that can be shed from the cell's surface as soluble CD44 (sCD44) in several body fluids, including FF. The administration of hCG may induce shedding of CD44 from granulosa cells and in fact FF sCD44 concentration varies according to the concentration of hCG. The concentration of sCD44 in FF was found to be lower in follicles containing oocytes that were subsequently fertilized and gave rise to good-quality embryos [105]. Whether or not high CD44 levels may directly adversely affect the oocyte is not known by now.

#### Alpha1-antitrypsin

Early studies indicated that follicles with mature oocytes contain lower levels of FF alpha-1-antitrypsin [106,107], therefore this protein could be a negative marker of oocyte quality. The research concerning the role of FF alpha-1-antitrypsin, however, has not been further developed in the last twenty years. It is unlikely that this substance will ever play a role in oocyte selection.

#### Leptin

FF leptin and soluble leptin receptor levels are inversely correlated with each other; FF leptin correlates directly with intrafollicular E2, P and androstenedione concentrations, whereas soluble leptin receptor levels are related to activin and follistatin intrafollicular concentrations [108]. Follicular leptin was also reported to inversely correlate with follicular pO2, appearing to be a marker of follicular hypoxia, suboptimal oocyte development [89] and low chance of pregnancy [109,110]. Other studies suggested that leptin levels in FF could not be considered a marker of oocyte quality and developmental potential [111]. In recent studies, leptin was shown to be positively correlated with the fertilization rate [112], weekly associated with embryo morphological score, but not with the IVF outcome [61]. At present, leptin concentration in FF does not appear to precisely reflect oocyte quality and to be useful for oocyte selection.

#### Endothelin-2

The mean concentration of endothelin-2 (but not endothelin-1) was reported to be significantly higher in FFs that contained oocytes that could be fertilized and cleave [113]. FF endothelin-2 levels were not correlated with inhibin, estradiol, progesterone or FSH, but significantly correlated with IGF-1 levels, indicating a possible synergistic effect with IGF-1 system in promoting FSH activity inside the follicle [113].

Plonowski [114] suggested that Endothelin-2 affects oocyte quality preventing the granulosa cells luteinization and progesterone synthesis; as a consequence, endothelin-2 could represent a positive marker of correctly-timed oocyte maturation.

#### Oocyte maturation inhibitor (OMI)

During normal follicle maturation it was observed a decline in FF OMI concentration as soon as the oocyte become mature and fertilizable. FFs yielding mature and fertilizable oocytes were found to have significantly lower OMI activity. Follicles with high OMI activity yielded immature or atretic oocytes, or oocytes with fractured zona pellucida [115]; it was postulated that in case of abnormal maturation or altered oocytes, FF OMI levels could remain higher and represent a negative marker of oocyte quality. However, OMI was the name attributed to an activity found in FF, but a corresponding protein was never identified. Thus, this paragraph has mainly an historical interest since research on OMI was not continued after the middle of 80's.

#### Homocysteine (HCY)

HCY is a metabolite of methionin with several functions not yet completely understood. It was observed that oocytes exposed to low HCY concentrations had better quality and degree of maturity [116,117]. Since administration of folic acid lowers HCY concentration in both FF and serum, this could explain the positive effect of pre-conceptional folic acid treatment on IVF outcome.

#### Beta-endorphin (beta-EP)

Data on the relationship between FF beta-EP and oocyte quality are controversial and inconclusive. Beta-endorphin levels were found to be higher in FF from follicles which contained oocytes that subsequently fertilized [118]. In contrast, another study observed that follicles containing mature oocytes had lower beta-EP content and were associated with oocytes able to form embryos with better developmental attitude [119].

#### Lactoferrin

Lactoferrin is an iron-binding glycoprotein originally isolated from milk. It is present in detectable amounts in FF and it was found to be significantly more concentrated in larger follicles yelding good quality oocytes able to allow fertilization and development to good quality embryos [120]. This recent observation needs to be further validated.

#### Angiotensin II (ATII)

FF ATII levels were found to be negatively correlated with FF progesterone [121]. However, another study found no correlation between ATII concentration in individual FFs and the ability of the corresponding oocyte to be fertilized [122].

#### Prorenin

Prorenin biosynthesis and secretion within the ovary are regulated by gonadotropins; higher prorenin FF levels are found in follicles containing immature oocytes, and no conception was noticed from oocytes originating from follicles with prorenin levels above the normal range [123]. Prorenin could be a negative marker of oocyte quality, but this hypothesis needs confirmation.

#### Amino-acids

Some amino-acids of FF, such as alanine and glycine, have shown a good predictive value on IVF outcome in the bovine model [124]. In humans, D-aspartic acid concentrations in FF were found to directly correlate with the percentage of good morphology, MII oocytes and with the fertilization rate [125]. This research area looks promising, but further, more complete data are needed.

### Sugars

#### Hyaluronan

Hyaluronan is a disaccharide composed of D-glucuronic acid and D-N-acetylglucosamine; it is contained in the cumulus extracellular matrix where it is involved in the detachment of the cumulus-oocyte complex that becomes freely floating in FF just before ovulation.

FF concentration of hyaluronan was positively related to the level of apoptosis in granulosa cells, and it was higher in follicles containing non-fertilized oocytes [126]. However, this observation was not confirmed and no significant relationship was found between FF hyaluronan and the maturity and fertilizability of the oocytes [127].

#### Myo-inositol

High levels of FF myo-inositol were positively correlated with FF E2 levels and with the quality of the embryos derived from the corresponding oocytes [128]. Myo-inositol, however, is not considered more reliable than E2 itself in indicating the quality of the oocyte.

### Prostanoids

Prostaglandin F2alpha, secreted by granulosa cells under the stimulation exerted by gonadotropins, was proposed as a biochemical marker of oocyte quality, as it was found to be higher in the FF of follicles whose oocytes were subsequently fertilized [6]. Moreover, both PGE2 and PGF2alpha concentration in FF were found to be higher in follicles containing mature oocytes [129].

In another study, the PGE2/PGF2alpha ratio in FF was found to be significantly lower in IVF cycles when pregnancy was obtained than in unsuccessful IVF cycles; this ratio could reflect the optimal conditions within the follicle to give an oocyte of high quality [130].

It must be remembered that prostaglandins have a very short half-life and their concentration in biological fluids changes very quickly: their measurement is technically difficult and not available in all laboratories. It is hard to imagine that prostanoids will be widely used as oocyte quality predictors in the clinical practice.

### Physicochemical features of FF

Apart from biochemical characteristics, the physicochemical properties of FF have been studied and correlated with the outcome of the corresponding oocytes.

The spectrophotometric absorbance of human FF in the visible spectrum shows two distinct peaks at 415 and 455 nm wavelength; it has been observed that oocytes that subsequently fertilized were more frequently associated with FFs having significantly higher absorbances at these two peaks [131,132]. Neither FF viscosity, or FF refractive index were found to correlate with oocyte maturity or fertilization potential [132].

### Metabolomics of FF

In the last years, research is moving away from metabolic target analysis (restricted to single metabolites) towards metabolite profiling (the study of selective groups of target compounds and their metabolic intermediates using a single analytical technique) and towards the dynamic study of all low molecular weight metabolites.

Metabolomics studies the small molecules (amino acids, lipids, nucleotides, signalling molecules, etc.) found in biological fluids that are produced through the action of different proteins, in turn expression of gene transcription and mRNA traduction. Metabolites represent the interface between the information embodied in the genome and the functional cell's phenotype. Their number is tipically lower than the number of genes and proteins in a cell or in an organism; as a consequence, thorough metabolomic analysis can be performed in a relatively faster time than genomic or proteomic analyses.

Metabolomics has been proven to be a consistent and informative technology for pattern recognition analysis of several biological systems, and is presently being applied to the study of human embryos [133-135] and oocytes [136]. Aim of metabolomic analysis is to identify and quantify all the metabolites in a biological fluid (e.g. FF) under given physiological conditions at a certain time point. The major difficulties in metabolomics are essentially three: a) many metabolites are of labile nature, chemically complex and have a widely dynamic production pattern; b) methods to amplify metabolites (e.g. as may be done with DNA) and increase sensitivity are lacking; c) metabolomic analysis deals at the same time with several classes of molecules with different chemical properties.

The techniques used in metabolomic studies have to be very sensitive and capable of screening a high number of molecules in a reasonably short time. At present, mass spectrometry (MS) techniques, alone or in combination with liquid chromatography, gas chromatography, capillary electrophoresis or ultra performance liquid chromatography (UPLC) offer the highest efficiency (for a review see [136]). The preliminary chromatographic step lowers the number of substances entering the mass spectrometer facilitating the separation of metabolites and improving MS data quality; integrated deconvolution algorhythms applied to informations coming from MS give a more precise quantification of metabolites [137,138]

Metabolomics of the FF is the dynamic quantitative assessment of all low molecular weight substances that are present in FF at a given time [139]. Being the end products of cell's metabolism, low-molecular weight metabolites can reveal the response of the follicle to all influences affecting its development. Metabolites are potentially more informative than the direct study of gene expression (genomics), mRNAs (transcriptomes) or proteins (proteomes); in fact, an increased gene activation with consequent mRNA and protein synthesis not necessarily corresponds to altered cellular function or morphology, whereas metabolomes reveal the actual functional status of a biological system and its cells.

Oocyte development is regulated by the transcripts accumulated during the growth phase; gene expression in oocytes may be studied by microarrays and high-fidelity RNA amplification techniques which allow the simultaneous profiling of thousands of genes, but cannot be used for oocyte selection because they require cell lysis [140]. Some reports describe RNA analysis of human oocytes using microarrays (transcriptomics) [141]. All mRNA molecules need to be translated into proteins or metabolites, that after specific post-translational modifications become the true markers of cell functionality. Metabolites represent the final products of cell regulatory processes; it is now possible to profile the metabolites secreted by the oocyte into the surrounding medium by investigating the FF or, alternatively, the compounds secreted by the oocyte into the culture medium in which it is suspended during in vitro culture after retrieval ("exometabolomics" or "secretomics"). The overall metabolic profiling of FF or of oocyte culture supernatants could be more useful than the targeted metabolic approach or the study of a selective class of substances for the assessment of oocyte quality. More than one metabolite, in fact, is likely to be involved in determining oocyte's developmental competence, and, as a consequence, a panel of biomarkers should be considered for clinical diagnostic purposes.

The most relevant investigations about the assessment of oocyte quality have been performed in animal models and have undertaken profiling for a selective class of metabolites, such as fatty acids [142], amino acids [143], or sugars [144]. Results are still very preliminary, but it has emerged that the metabolic profiling of FF collected from large antral follicles is more homogeneous that the one obtained with fluids collected from small follicles, reflecting differences in the biochemical profile linked to oocyte maturational stage [145]. In one of these studies, it has been observed that oocytes able to absorb larger amounts of glucose and actively convert it into lactate show the highest fertilization potential [144]. Other studies have focused on the evaluation of oocyte metabolism through the measurement of energy substrates [146] or of oxygen consumption [147] in culture media. The concentration of oxygen in the medium containing the oocyte may be measured by specific and sensitive sensors; it is assumed to reflect the mitochondrial activity necessary to produce the energy required for development, and it has been shown to be related to cell's viability [147].

Overall, the viability and quality of oocytes (and derived embryos) estabilished by metabolomic tests could represent stronger predictors for implantation potential than the classical morphological assessment. Maybe the metabolomic analysis of the FF collected at OPU may provide additional predictive informations about egg quality in the next future. The predictive value of metabolomic analysis will be fully realized when new technologies, able to determine a broadest range of metabolites by a single analytical platform, will be developed. Large-scale, prospective studies are required to relate the metabolic profile of FF to the developmental potential of human oocytes and to apply this non-invasive technology to clinics.

## Conclusion

Ideally FF, a superfluous, abundant and easily available material in IVF cycles, would be an optimal source on non-invasive predictors of oocyte quality. Unfortunately, however, most studies aiming to find a good molecular predictor of oocyte quality in FF are mainly correlative and not performed on large-scale, prospective and well-controlled basis. Furthermore, most of them analyze the correlation between specific molecules and the oocyte performance using univariate, and not multivariate, analysis algorhythms. As a consequence, until now no single substance has been identified as a reliable marker of oocyte competence to fertilization, embryo development and pregnancy.

The metabolomic approach is a powerful tool to study such marker(s) in FF, but its application is still at the beginning; this techniques is facing the problems arising from analysing a complex biological fluid such as FF.

Hopefully integrated criteria, taking together oocyte morphology, molecular predictors in FF and data about gene espression in granulosa cells, would give more precise information and will help embryologists to achieve a better capability of selecting optimal quality, "golden" oocytes in the next years.

## Competing interests

The authors declare that they have no competing interests.

## Authors' contributions

All authors contributed to bibliographic research, critical evaluation of the articles included in the manuscript and writing of the manuscript. PR was also in charge of the final language revision. All authors read and approved the final manuscript.
